# Alexithymia and Somatization in Chronic Pain Patients: A Sequential Mediation Model

**DOI:** 10.3389/fpsyg.2020.545881

**Published:** 2020-10-27

**Authors:** Roberta Lanzara, Chiara Conti, Martina Camelio, Paolo Cannizzaro, Vittorio Lalli, Rosa Grazia Bellomo, Raoul Saggini, Piero Porcelli

**Affiliations:** ^1^Department of Dynamic and Clinical Psychology, “Sapienza” University of Rome, Rome, Italy; ^2^Department of Psychological, Health and Territorial Sciences, University “G. d’Annunzio” of Chieti-Pescara, Chieti, Italy; ^3^Department of Anesthesia and Intensive Care, Regional Pain Unit, University Hospital SS. Annunziata, Chieti, Italy; ^4^Department of Biomolecular Sciences, University of Urbino Carlo Bo, Urbino, Italy; ^5^Department of Medical, Oral and Biotechnological Sciences, University “G. d’Annunzio”, Chieti, Italy

**Keywords:** chronic pain, alexithymia, somatization, distress, quality of life

## Abstract

**Objective:**

To investigate whether chronic pain (CP) patients with somatization reported higher alexithymic traits than those without somatization and to study the different relationships between psychological characteristics, pain, health-related quality of life (HRQL), and somatization.

**Method:**

A consecutive sample of 134 CP treatment-seeking outpatients were evaluated for alexithymia (TAS-20), somatization (PHQ-15), distress (HADS), HRQL (SF-12), and pain (BPI).

**Results:**

Patients with somatization (37.04%) reported significantly higher TAS-20 total scores (*p* < 0.001) and difficulty in identifying feelings (DIF) (*p* < 0.001) than those without somatization. The somatizer group had also a significantly higher disease duration, severity and interference of pain, distress, and lower HRQL than the non-somatizer group. Hierarchical regression analysis showed that although distress, pain interference and the mental HRQL component are closely related to somatization (*R*^2^ = 0.55), DIF was the strongest predictor of severity of somatization (β = 0.31). A sequential indirect effect from DIF to somatization via distress symptoms and pain interference turned out to be significant [95% CI (0.01, 0.09)]. Support was also found for sequential mediation paths from DIF to somatization via distress and mental HRQL [95% CI (0.01, 0.11)].

**Conclusions:**

Our results pointed-out that alexithymia, particularly DIF, may be major factor for somatization risk in CP patients. Longitudinal observations are needed for evaluating the role of alexithymia in clinical outcomes.

## Introduction

Chronic pain (CP) is a common, heterogeneous, and distressing problem, which has a significant impact on society and individuals (Fayaz et al., 2016). In Europe, approximately 19% of adults experience CP (Breivik et al., 2006), which affects well-being and healthcare use (Reid et al., 2011). This involves about 200 million people suffering from musculoskeletal disorders, 153 million people experiencing a migraine or other disabling headaches, and 100 million people suffering from other forms of CP (Breivik et al., 2006; Breivik et al., 2013). The International Association for the Study of Pain (IASP) defines CP as pain which has persisted for more than 3 months or beyond the expected time for healing (Merskey and Bogduk, 1994). Unlike acute pain, CP originates from an organic condition no longer solvable and has broad social and economic implications (International Association for the Study of Pain, 2012). In Europe, the annually estimated direct and indirect healthcare costs for CP would result in about 440 billion euros (Societal Impact of Pain, 2017). In the United States, the estimated economic burden of treatment and lost productivity resulting from CP is 560–635 billion dollars per year, but for many people, pain treatment is not fitting (Institute of Medicine, 2011).

The nature of CP reflects extremely various epidemiology that complicates the development of adequate solutions, with sever repercussions in the individual quality of life. Patients with CP are often at risk for developing further complications, including depressive symptoms, disability, conflicted relationship, drug dependence, and even death (Vowles et al., 2015; Morales-Espinoza et al., 2016). Although treatments for CP include pharmacological, procedural (e.g., injections), or surgical interventions, patients are often not satisfied with the results, costs, and adverse effects (Deyo et al., 2009; Babatunde et al., 2017; Wiffen and Xia, 2020). An overview of reviews summarizing the evidence on currently available treatment options for musculoskeletal pain found that non-steroidal anti-inflammatory drugs had short-term efficacy, with moderate evidence (*d* ≤ 0.50) and a high potential for adverse effects. Corticosteroid injections had positive effects for the short-term, but recent evidence remains unclear on an optimal dose, intensity, and frequency, or mode of application (Babatunde et al., 2017). Furthermore, often patient illness behaviors differ from physicians’ recommendations, and the greater is the discrepancy, the less is likely the course of the disease will be predicted solely by biomedical factors (Sirri et al., 2013). Currently, CP is conceived as the final common pathway of complex interactions between the central nervous system and lifelong neurophysiological and psychological events and experiences (Gatchel et al., 2007; Apkarian et al., 2009, 2013), challenging classical biomedical models. A biopsychosocial approach is considered the most heuristic perspective to the understanding and treatment of CP (Gatchel et al., 2007). For these reasons, there is an urgent need to explore the possibility that psychological processes can affect the CP response.

The diagnosis and clinical treatment of CP are particularly challenging due to their overlap with somatization, the tendency to experience and report somatic symptoms due to psychological distress (Lipowski, 1988). Given the uncertainty in differentiating pain and clinically relevant somatization (generally diagnosed as a Somatic Symptom Disorder [SSD] and, previously, as Somatoform Disorder [SD]), there is relatively sparse research on SSD and SD in CP patients. For example, in a review of the literature (Birket-Smith, 2001), the frequency of SD in CP patients varied from 0 to 53%. This discrepancy may be due to the inherent problems in the pain assessment. Nevertheless, studies that measure both pain and somatization describe significant correlations between the two constructs (Fishbain et al., 2009). Similar theoretical etiologies have been described to underlie both pain and somatization, such as neurobiological sensitivity and/or central sensitization (Bourke et al., 2015; Adams and Turk, 2018), cognitive-affective mechanisms (Asmundson et al., 2012), and social learning and familial transmission (Rousseau et al., 2014; Stone and Wilson, 2016). However, the exact relationship between pain and somatization is not clear. Some researchers describe certain pain conditions as examples of somatic symptoms (Genizi et al., 2013; Ratnamohan and Kozlowska, 2017), while others suggest that there is evidence for the role of somatization in the evolution from acute pain to CP (McBeth et al., 2001; Pincus et al., 2002; Clark et al., 2017). For example, some studies found somatization is confirmed as having a role in the progression to chronicity in low back pain. Nevertheless, their effect size varied substantially, ranging from *d* = 0.2 (Dionne et al., 1995) to *d* = 0.6 at 1-year follow-up (Burton et al., 1995), and *d* = 0.9 at 2-year follow-up (Dionne et al., 1997). Overall, the co-occurrence of pain and somatization is related to numerous negative functional outcomes (Ailliet et al., 2016; Eliasen et al., 2016).

A factor potentially involved both in somatization and CP is alexithymia, a multidimensional construct characterized by deficits in the cognitive processing of emotions. It is widely recognized that alexithymia affects health in several psychiatric (Lumley et al., 2007) and medical (Porcelli et al., 2013) disorders. Given that alexithymia is thought to reflect deficits in mentalizing emotions, individuals with high levels of alexithymia may be more prone to experiencing poorly differentiated negative emotions and their somatic manifestations (Luminet et al., 2018). Influencing illness perception and behavior (including response to treatment), it is not surprising that CP patients have been reported with high levels of alexithymia (Di Tella and Castelli, 2016). Several studies have found that is mainly the difficulty in identifying feelings (DIF) trait of alexithymia that is linked both with the tendency to somatize (e.g., De Gucht et al., 2004; Bailey and Henry, 2007; Mattila et al., 2008) and CP (for a review, see Di Tella and Castelli, 2016). Despite several studies found a positive association between alexithymia and somatization (Porcelli and Taylor, 2018), they generally failed to control for negative affectivity. Other studies have also found that the association between alexithymia and somatization became no significant after controlling for distress (De Gucht and Heiser, 2003; Bailey and Henry, 2007; Mattila et al., 2008).

The association between CP, somatization, distress, and alexithymia is particularly relevant. Several factors, not fully understood, may explain this link. For example, the central sensitization mechanism in CP is an emotional disorder that produces a dysregulation in pain perception, mainly on its affective component (Nijs et al., 2010; Arnold et al., 2016). Studies that considered the difference between sensory and affective components of pain have reported that alexithymia was related to the affective dimension of pain more than sensory pain (Sancassiani et al., 2017). In particular, the literature shows that alexithymia is significantly associated with worse perceptions of mental health-related quality of life (HRQL) and more pain interference than physical HRQL and pain severity (Di Tella and Castelli, 2016; Horta-Baas et al., 2020). The evidence available suggests a possible role of distress in mediating the relationship between alexithymia, HRQL (Castelli et al., 2012; Tesio et al., 2018; Larice et al., 2020) and pain disability (Saariaho et al., 2013; Di Tella et al., 2017) in CP patients. Distress symptoms may interfere with the alexithymic patient’s capacity to cope with pain, worsening the patients’ HRQL and leading them to focus on the somatic sensations (Lumley et al., 1996, 2007).

To our knowledge, no studies investigated the relationship between alexithymia and somatization in CP populations, considering the role of distress, perception of pain, and HRQL. Therefore, this study aimed: (a) to confirm whether CP patients with somatization reported higher alexithymic traits than those without somatization; (b) to investigate the independent role of each alexithymia dimension to predict somatization in CP patients; (c) to test a theoretical model which assumed that distress, pain interference, and mental HRQL sequentially mediate the relationship between DIF and somatization among CP patients. We expected that: (a) patients with somatization would exhibit more alexithymia than non-somatizer CP patients; (b) DIF would be more predictive of somatization than other alexithymia dimensions in CP patients; (c) DIF would affect somatization both directly and through the mediating role of distress symptoms, pain interference, and mental HRQL.

## Materials and Methods

### Participants

A sample of 134 treatment-seeking CP outpatients was consecutively recruited at their first visit at the Pain Unit at the University Clinical Hospital of Chieti (Italy). Patients were first screened for medical and psychological conditions. All participants have been diagnosed with CP by expert medical specialists. Non-malignant CP lasting for 3 months or more was considered inclusion criteria (International Association for the Study of Pain, 1994). To improve ecological validity, we included all adult outpatients from 18 to 70 years old. Self-reported major psychiatric disorders, pregnancy, impairment in cognitive functions, inability to perform or understand the self-administered questionnaires, documented cancer pain or acute pain diagnosis, were considered exclusion criteria.

The subjects involved in the study gave written informed consent to participate. The study was realized following the World Medical Association Declaration of Helsinki and its subsequent revisions (General Assembly of the World Medical Association, 2014) and approved by the local Ethics Committee.

### Measures

#### Socio-Demographic and Clinical Variables

The socio-demographic characteristics such as age, gender, years of education, and marital status were collected using an *ad hoc* semi-structured interview. The disease duration was calculated as the time of onset of their self-reported first symptoms.

#### Pain Evaluation

The Brief Pain Inventory (BPI) (Cleeland and Ryan, 1991) was used to assess the level of pain. The BPI is a self-administered questionnaire used for assessing clinical pain and includes two main subscales for pain severity (PS) (4-items) and pain interference (PI) (7-items). For BPI-PS, items are rated on a 10-point Likert scale ranging from (= no pain) to 10 (= pain as bad as you can imagine), and contributes with the same weight to the final score, ranging from 0 to 40. For BPI-PI, items are rated on a 10-point Likert scale ranging from 0 (= does not interfere) to 10 (= completely interferes), and contributes with the same weight to the final score, ranging from 0 to 70. For our sample, Cronbach’s α was 0.89 for the severity and 0.90 for the interference subscales.

#### Tendency to Somatization

The self-rating 15-item Patient Health Questionnaire (PHQ-15) (Kroenke et al., 2002) was used to assess the tendency to somatization. The PHQ-15 is widely used as a screening measure for somatization in various medical settings. Patients were asked for the last 1-month to rate the severity of 15 symptoms as 0 (= not bothered at all), 1 (= bothered a little), or 2 (= bothered a lot), with total scores ranging from 0 to 30 and scores of ≥ 5, ≥ 10, ≥ 15 represent low, moderate and severe levels of somatization. For this sample, Cronbach’s α was 0.82.

#### Alexithymia

The self-report 20-item Toronto Alexithymia Scale (TAS-20) (Bagby et al., 1994) was used to measure alexithymia. Each item is scored on a 5-point Likert scale ranging from 1 (= strongly disagree) to 5 (= strongly agree), with total scores ranging from 20 to 100, and scores of ≥ 61 represent the threshold for higher levels of alexithymia (Taylor et al., 1997). The TAS-20 produces three subscales to assess the difficulty in identifying feelings (DIF), the difficulty in describing feelings (DDF) to other people, and externally oriented thinking (EOT). The TAS-20 demonstrates good test-retest reliability, internal consistency, construct validity, and factor structure (Bressi et al., 1996; Bagby et al., 2020). For our sample, Cronbach’s α was 0.81 for the total scale, 0.77 for the DIF, 0.70 for the DDF, and 0.57 for the EOT subscales.

#### Emotional Distress

The depressive and anxiety symptoms were evaluated through the Hospital Anxiety and Depression Scale (HADS) (Zigmond and Snaith, 1983; Bjelland et al., 2002), a self-administered questionnaire used for assessing emotional distress in patients with physical health problems. The HADS gives two main scores for depressive (7-items) and anxiety (7-items) symptoms. Each item is rated from 0 (= no symptom) to 3 (= definite experience of symptoms). For each subscale, scores ranging between 8 to 10 are considered as borderline and from 11 to 21 as moderate to severe symptoms of anxiety and depression. The HADS has been used in different health care settings, demonstrating good reliability and validity (Norton et al., 2013). In our sample, Cronbach’s α was 0.82 for the total scale, 0.83 for anxiety, and 0.62 for depression subscales.

#### Health-Related Quality of Life

The 12-Item Short Form Health Survey (SF-12) (Gandek et al., 1998) was used to evaluate HRQL. It is a generic questionnaire related to functioning and well-being in physical, mental, and social dimensions of life. The SF-12 includes two subscales, the Physical Component Summary (PCS-12) and the Mental Component Summary (MCS-12). Each subscale consists of 12-items whose scores are transformed to produce a total score ranging from 0 to 100, considering HRQL to be better when the score is higher. For this sample, Cronbach’s α was 0.61 for PCS-12 and 0.59 for MCS-12 dimensions.

### Statistical Analysis

For statistical analysis, a 3-step strategy was performed.

Firstly, socio-demographic, clinical, and psychological variables between CP patients with low and high levels of somatization were compared using Student’s *t*-tests or chi-square test (χ^2^). The standardized mean difference was used as a measure of effect size (Cohen’s *d*) and provides three magnitudes of effects of 0.20–0.50, 0.50–0.80, and >0.80 represent small, moderate, and large effects size (Cohen, 1988). The Eta-squared (η*^2^*), a measure of effect size for χ^2^, was also used. A standardized effect size (η*^2^*) of 0.01–0.05 is considered small, 0.06–0.14 moderate, and >0.14 large. Pearson’s correlation coefficient was performed to evaluate the relationships between clinical and psychological variables.

Secondly, hierarchical regression analysis was performed to point out major factors that best predict somatization. Somatization was considered as a dependent variable and the independent variables were socio-demographic characteristics, TAS-20 dimensions, HADS total score, SF-12 dimensions, and BPI-PS, and BPI-PI. Four regression models were estimated, and regression coefficients and the corresponding *p* values were calculated. In the first model, the socio-demographic characteristics were entered, and in the three following models, the added variables potentially explaining the outcome were forced in. To evaluate the contribution of alexithymia to somatization before adjusting for other clinical variables, we included the TAS-20 dimensions in the second model. Specifically, we aimed to test the extent to which each variable (i.e., TAS-20 dimensions in the second model, HADS total score and SF-12 dimensions in the third model, and BPI-PS and BPI-PI in the fourth model) were able to significantly add to the final explained variance of somatization.

Thirdly, according to the previous analysis, we performed a path sequential multiple mediational models to assess the effect of DIF on the severity of somatization through the mediating role of HADS total score, BPI-PI, and MCS-12. In the performed model, three mediators intervene in a series between an independent and a dependent variable (Taylor et al., 2008). To analyze the model, four regression equations were estimated: (a) regressing mediator 1 (HADS total score) on the independent variable (DIF), (b) regressing mediator 2 (BPI-PI) on mediator 1 (HADS total score) and independent variable (DIF), (c) regressing mediator 3 (MCS-12) on mediator 1 (HADS total score), mediator 2 (BPI-PI), and independent variable (DIF), (d) regressing the dependent variable (somatization) on mediator 1 (HADS total score), mediator 2 (BPI-PI), mediator 3 (MCS-12) and independent variable (DIF). In all four regression equations, gender was included as a covariate.

All statistical analyses were computed using IBM SPSS Statistics version 25 (IBM Corp., Armonk, NY). The tested mediation model was estimated using PROCESS macro for SPSS (Hayes, 2013), according to ordinary least-squares (OLS) regression (Model 6). The bootstrapping procedure was used for testing the significance of the indirect effects (Preacher and Hayes, 2008). We apply 5,000 bootstraps resample to evaluate the bias-corrected 95% confidence interval (CI). When the interval did not contain zero, the effect was considered statistically significant at *p* < 0.05.

## Results

### Characteristics of the Sample

The socio-demographic and clinical variables of the total sample are shown in Table 1.

**TABLE 1:** Comparisons of socio-demographic and clinical characteristics between patients with absent-to-low and moderate-to-high somatization.

Variables		Total sample *N* = 134	Absent-low Somatization *N* = 85 (62.96%)	High-moderate Somatization *N* = 49 (37.04%)	t/χ2	*p*	*d/*η*^2^*
Age *(M* ± *SD)*		49.96 ± 17.02	48.61 ± 17.84	52.33 ± 15.37	1.21	0.22	0.22
Gender	Male	60 (44.77)	43 (50.59)	17 (34.69)	3.17	0.10	0.15
	Female	74 (55.23)	42 (49.41)	32 (65.31)			
Education		12.44 ± 4	12.68 ± 3.99	11.98 ± 4.02	0.92	0.35	0.18
Marital status	Unmarried	41 (30.60)	29 (34.12)	12 (24.49)	1.64	0.80	0.11
	Currently married	91 (67.91)	56 (65.88)	37 (75.51)			
Disease duration		6.98 ± 9.28	5.47 ± 7.67	9.60 ± 11.16	2.53	0.01	0.46
TAS-20 tot (*M* ± *SD)*		45.24 ± 13.16	41.44 ± 11.48	51.84 ± 13.38	4.74	<0.001	0.86
DIF (*M* ± *SD)*		15.29 ± 6.58	12.81 ± 5.20	19.59 ± 6.56	6.58	<0.001	1.19
DDF (*M* ± *SD)*		12.87 ± 3.47	12.47 ± 3.19	13.57 ± 3.84	1.78	0.07	0.32
EOT (*M* ± *SD)*		19.10 ± 5.56	18.67 ± 5.43	19.86 ± 5.76	1.19	0.23	0.22
BPI-PS (*M* ± *SD)*		19.04 ± 9.08	17.00 ± 8.70	22.59 ± 8.70	3.58	<0.001	0.65
BPI-PI (*M* ± *SD)*		37.22 ± 16.65	32.67 ± 15.65	45.10 ± 15.49	4.44	<0.001	0.80
HADS tot (*M* ± *SD)*		15.60 ± 7.98	12.78 ± 6.56	20.51 ± 7.91	6.08	<0.001	1.10
PCS-12 (*M* ± *SD)*		36.98 ± 11.10	38.17 ± 11.52	34.69 ± 10.07	1.76	0.08	0.32
MCS-12 (*M* ± *SD)*		45.37 ± 11.66	49.12 ± 10.13	38.86 ± 11.37	5.39	<0.001	0.98

*BPI-PS, Bref Pain Inventory–Pain Severity; BPI-PI, Bref Pain Inventory–Pain Interference; DIF, Difficulty in Identifying Feelings; DDF, Difficulty Describing Feelings; EOT, Externally Oriented Thinking; HADS tot = Hospital Anxiety and Depression Scale Total Score; MCS-12, Mental Component Summary-12; PCS-12, Physical component summary-12; TAS-20 tot, Toronto Alexithymia Scale-20 Total Score.*

Of the 157 recruited patients, 134 (85.35%) were studied. Lack of time was the main reason for not participating. Enrolled patients reported a mean age of 49.96 ± 17.02 years and a mean education of 12.44 ± 4 years. Most of the participants were women (*n* = 74, 55.22%) and married (*n* = 91, 68.94%). According to PHQ-15 thresholds (see section “Tendency to Somatization”), 37.04% (*n* = 49) of CP patients showed a clinically significant tendency to somatization.

### Between-Group Comparisons

The comparisons between patients with low and high levels of somatization are shown in Table 1.

Patients with high levels of somatization reported significantly higher disease duration (9.60 ± 11.16 vs. 5.47 ± 7.67; *t* = 2.53, *p* < 0.01), although with a small effect size (*d* = 0.46). No sociodemographic differences were found between the two groups. Alexithymia, psychological distress, pain, and HRQL showed significant between-group differences, with effect sizes in the moderate and high ranges. Specifically, high-level somatization patients scored significantly higher to TAS-20 (*d* = 0.86), DIF (*d* = 1.19), BPI-PS (*d* = 0.65), BPI-PI (*d* = 0.80), HADS (*d* = 1.10) and MCS-12 (*d* = 0.98) scores than those with low levels of somatization.

### Between-Variable Associations

Table 2 reports zero-order correlations between the psychological variables of the study sample.

**TABLE 2:** Zero-order correlations between psychological variables of the study sample.

	1	2	3	4	5	6	7	8	9	10	11
1. Disease duration	1.00										
2. TAS-20 tot	0.136	1.00									
3. DIF	0.142	0.833***	1.00								
4. DDF	0.070	0.646***	0.443***	1.00							
5. EOT	0.115	0.752***	0.353***	0.455***	1.00						
6. BPI-PS	0.246**	0.148	0.220*	0.058	0.113	1.00					
7. BP-PI	0.286**	0.296**	0.392***	0.120	0.132	0.633***	1.00				
8. HADS tot	0.144	0.431***	0.568***	0.138	0.133	0.332***	0.526***	1.00			
9. PCS-12	–0.149	0.030	–0.038	0.063	0.045	−0.297**	−0.414***	−0.231**	1.00		
10. MCS-12	–0.023	−0.336***	−0.442**	–0.162	–0.077	–0.157	−0.295**	−0.608***	0.211*	1.00	
11. PHQ-15	0.168	0.412***	0.568***	0.143	0.109	0.403***	0.517***	0.593***	−0.290**	−0.494***	1.00

BPI-PS, Bref Pain Inventory–Pain Severity; BPI-PI, Bref Pain Inventory–Pain Interference; DIF, Difficulty in Identifying Feelings; DDF, Difficulty Describing Feelings; EOT, Externally Oriented Thinking; HADS tot, Hospital Anxiety and Depression Scale Total Notes: Score, MCS-12, Mental Component Summary-12; PCS-12, Physical component summary-12; PHQ-15, Patient Health Questionnaire-15; TAS-20 tot, Toronto Alexithymia Scale-20 Total Score. All value are standardized regression weights. *p < 0.05; **p < 0.01; ***p < 0.001.

Multiple significant correlations in the moderate and high ranges (*r* > 0.30) were found among variables. Somatization was significantly associated with alexithymia, pain, psychological distress, and HRQL. In particular, the PHQ-15 correlated higher with TAS-20 (*r* = 0.41), DIF (*r* = 0.56), BPI-PS (*r* = 0.40), BPI-PI (*r* = 0.51), HADS (*r* = 0.59), MCS-12 (*r* = −0.49) scores, and at a lower level with PCS-12 score (*r* = −0.29).

Table 3 reports the hierarchical regression model with somatization as an outcome variable.

**TABLE 3:** Hierarchical regression model predicting somatization in CP patients.

	β	t	*p*	R	R^2^
**Model 1**				0.24	0.05
Age	0.07	0.79	0.43		
Gender	0.20	2.26	0.02		
Education	–0.05	–0.57	0.57		
**Model 2**				0.62	0.38
Age	0.05	0.74	0.45		
Gender	0.15	2.25	0.02		
Education	0.02	0.32	0.74		
DIF	0.61	7.48	<0.001		
DDF	–0.05	–0.59	0.55		
EOT	–0.08	–0.92	0.35		
**Model 3**				0.72	0.52
Age	0.05	0.74	0.45		
Gender	0.15	2.25	0.02		
Education	–0.01	–0.24	0.81		
DIF	0.35	3.81	<0.001		
DDF	–0.01	–0.09	0.92		
EOT	–0.06	–0.76	0.44		
HADS tot	0.19	1.96	0.05		
PCS-12	–0.16	–2.29	0.02		
MCS-12	–0.23	–2.64	0.009		
**Model 4**				0.74	0.55
Age	–0.03	–0.45	0.65		
Gender	0.13	1.96	0.05		
Education	0.02	0.30	0.76		
DIF	0.31	3.46	0.001		
DDF	–0.02	–0.36	0.71		
EOT	–0.03	–0.44	0.65		
HADS-tot	0.11	1.13	0.26		
PCS-12	–0.10	–1.48	0.13		
MCS-12	–0.22	–2.67	0.009		
BPI-PS	0.14	1.63	0.10		
BPI-PI	0.18	1.79	0.05		

*BPI-PS, Bref Pain Inventory–Pain Severity; BPI-PI, Bref Pain Inventory–Pain Interference; DIF, Difficulty in Identifying Feelings; DDF, Difficulty Describing Feelings; EOT, Externally Oriented Thinking; HADS tot, Hospital Anxiety and Depression Scale Total Score; MCS-12, Mental Component Summary-12; PCS-12, Physical component summary-12; PHQ-15, Patient Health Questionnaire-15; TAS-20 tot, Toronto Alexithymia Scale-20 Total Score.*

In the first model, sociodemographic characteristics (age, gender, and years of education) explained 0.5% of somatization, with the only gender showing the greater β of 1.96 (*t* = 2.26; *p* = 0.02). Adding TAS-20 subscales produced an added predictor of somatization of 33% (Model 2), with the only DIF showing the greater β of 0.61 (*t* = 7.48; *p* = < 0.001). When HADS (β = 0.19; *t* = 1.96; *p* = 0.05), PCS-12 (β = −0.16; *t* = −2.29; *p* = 0.02) and MCS-12 (β = −0.23; *t* = −2.64; *p* = 0.009) scores were added in Model 3, they significantly explained an added 14% of somatization variance, that was increased by 3% when BPI-PS and BPI-PI were included as predictor in Model 4 (β = 0.14; *t* = 1.63; *p* = 0.10 and β = 0.18; *t* = 1.79; *p* = 0.05, respectively). The final model predicted 55% of the explained variance, with gender (*p* = 0.05) and DIF (*p* = 0.001), MCS-12 (*p* = 0.009) and BPI-PI (*p* = 0.05) scores were the main predictors for somatization.

### Sequential Mediation Model

**FIGURE 1:**
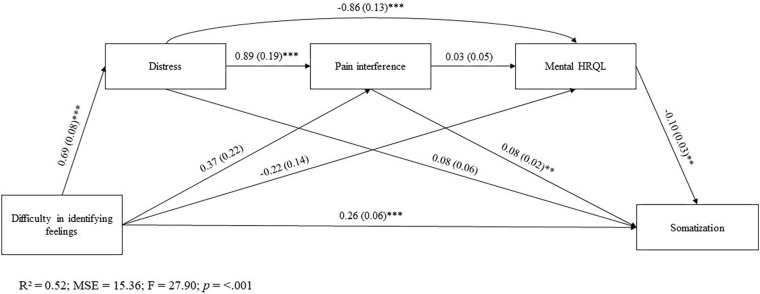
All value are standardized regression weights. *p < 0.05; **p < 0.01; ***p < 0.001. R^2^, R-squared; MSE, Mean Squared Error; F, F-distribution.

Figure 1 reports the results of the sequential mediation analysis.

After accounting for gender factor, most of the direct effects between the key variables in the model result to be significant, except for following individual paths: (a) DIF on BPI-PI and MCS-12 scores; (b) BPI-PI on MCS-12 score; and (c) HADS on PHQ-15 score.

The mediation analysis showed that DIF affected somatization along different pathways. There was a significant sequential indirect effect of DIF on somatization through distress and pain interference [β = 0.05, SE = 0.02; 95%CI(0.01, 0.09)]. Moreover, significant were sequential mediation paths from DIF to somatization via distress and mental HRQL [β = 0.02, SE = 0.02; 95%CI(0.01, 0.11)].

## Discussion

Chronic pain is recognized as a multifaceted experience made up of a dynamic interaction of sensory, affective, and cognitive factors, in which biopsychosocial components interact with each other in defining the clinical expression of pain (Gagliese and Katz, 2012). There are a longstanding public and scientific interest in debating how personality dimensions (such as alexithymia) and psychological or psychopathological factors (such as the tendency to somatization) influence the diagnosis and trajectory of CP disorders (Friedrichsdorf et al., 2016; Naylor et al., 2017). Several studies showed evidence of a robust association of alexithymia with somatization (Porcelli and Taylor, 2018). Even though alexithymia (Aaron et al., 2019) and somatization (McAndrew et al., 2019) have been demonstrated to be related to pain and pain-related functioning in patients with CP, the relationship between these factors in CP patients are not fully understood. To date, only a few studies examined the relationship between alexithymia and somatization in CP populations. For example, a Turkish study (Tuzer et al., 2011) found that alexithymia was a risk factor for incorrectly attributing common physical symptoms to physical disease in chronic low back pain patients. Therefore, analyzing the role of somatization and alexithymia could shed light on the personality profile of CP patients.

As expected in our first and second hypotheses, CP patients with higher levels of somatization had high alexithymia than those with lower levels of somatization. More specifically, DIF appeared as the only alexithymia factor significantly associated with somatization and was the major predictor of somatization within our sample, even after adjustment for all sociodemographic, emotional distress, pain-related, and HRQL factors. Our findings suggest that alexithymia, particularly the facet of DIF, was more prominent in explaining somatization symptoms in CP patients. Literature shows that somatization is related to a restricted ability to consciously experience and recognize emotions and explicit them adequately (Waller and Sheidt, 2006). As suggested, alexithymia can contribute to somatic symptoms formation through sustained physiological arousal that may lead to altered system functioning and tissue damage (e.g., vulnerability to inflammatory processes) (Luminet et al., 2018). Another pathway is that the difficulties in affect awareness may impair the ability to modulate emotions, leading to a focus on and increase of bodily states associated with emotional activation, and potential misinterpretation of those states as characterizing bodily illness. Therefore, somatization could be appeared as deriving from alexithymia, as the effort in elaborating emotional states in symbolic form leaves the individual with an unsymbolized bodily expression of emotion (Bucci, 1997). In line with studies that found positive correlations between DIF and somatization (for a review see Taylor et al., 1997), our results showed that DIF was a strong independent predictor of increased somatization, even after adjustment for distress symptoms. On the contrary, some studies found that the association was no longer significant after controlling for psychological distress (De Gucht and Heiser, 2003; Bailey and Henry, 2007; Mattila et al., 2008). Exploring the relationship between alexithymia and somatization is particularly difficult since both constructs are closely related to negative affectivity, such as the tendency to experience emotional and somatic distress (Porcelli and Taylor, 2018).

In our third hypothesis, DIF was expected it would affect somatization both directly and through the relevant contribution of emotional distress, pain interference and mental HRQL as mediating factors. This hypothesis was fully confirmed. Our results pointed out that the effect of DIF on somatization may occur both directly and above all through the mediation of emotional distress. From one side, emotional distress may predispose alexithymic individuals to experience high pain interference. From the other side, distress may induce alexithymic individuals to experience a low mental and social HRQL. The DIF dimension is directly related to somatization, while the effect of DIF is mediated by emotional distress when also the pain interference and the mental HRQL component are considered. Consistently, several longitudinal studies have demonstrated that alexithymia and emotional distress are connected with many factors in the emotional and cognitive dimensions of the pain experience, which have been shown to have an important role in the development and maintenance of CP (Saariaho et al., 2016, 2017). For example, Saariaho et al. (2017) studied the impact of alexithymia, depressive symptoms, baseline pain levels, and treatment options on the course of CP in a clinical sample. The results showed that baseline alexithymia was related with more pain disability, depression, and more use of opioids after a 1-year follow-up. Emotional distress and alexithymia (particularly difficulty in identifying emotional states) are likely to induce high levels of somatic symptoms reported as adding factor to CP impairment (Keefe et al., 2004; Flor and Turk, 2011; Vlayen and Linton, 2012; Bean et al., 2014; Martínez et al., 2015). Literature shows that alexithymia and distress are associated with several psychosocial factors persisting and exacerbating the pain status, causing an increase of pain over-treatment, problematic CP clinical management, and increased healthcare costs (Oberleitner et al., 2019).

The results of our study should be assumed considering some limitations. First, we did not include any control groups, studies should investigate whether the role of alexithymia is different in patients with acute pain or other chronic conditions. Second, the sample size was relatively small, so that it may not be representative of all CP patients. Third, alexithymia was evaluated with a self-administered questionnaire only while a multimethod assessment approach involving structured interviews (Bagby et al., 2006) should be used in future studies. Fourth, the cross-sectional nature of this study does not allow us to determine the predictive effect of DIF on the development and persistence of somatization symptoms over time, because of only a longitudinal design may help to explain this point. Moreover, longitudinal studies are needed to investigate the causal relationship between alexithymia, distress, and somatization.

Considering these limitations, CP patients who present somatic symptoms should be screened for their level of alexithymia and emotional distress, given that the difficulty in awareness of feelings is likely to influence the clinical manifestation and management of pain. Alexithymia, specifically DIF, might induce a somatic amplification of pain and increase somatic symptoms reporting as adding factor to adverse effects of CP treatments (Lumley et al., 2007; Honkalampi et al., 2011). The alexithymic tendency to emphasizing physical symptoms rather than emotional states could influence the treatment decisions and induce to fail the assessment of distress symptoms. There is evidence that alexithymia can be successfully reduced with therapeutic interventions (Cameron et al., 2014). The treatment of alexithymia may show high benefit for the perceived HRQL of medical patients (Ogrodniczuk et al., 2011) and may significantly reduce pain (Tulipani et al., 2010; Porcelli et al., 2011). Tailored interventions on specific features of CP patients as the tendency to somatization, difficulty in the cognitive processing of emotions and distress should be carefully assessed by clinicians to improve the therapeutic effectiveness of treatment interventions.

## Data Availability Statement

The raw data supporting the conclusions of this article will be made available by the authors, without undue reservation.

## Ethics Statement

The studies involving human participants were reviewed and approved by Ethics Committee of University G.d’Annunzio – Chieti-Pescara. The patients/participants provided their written informed consent to participate in this study.

## Author Contributions

RL and CC wrote the manuscript, conducted statistical analysis, and provided substantial contributions to the conception and design of the manuscript. MC wrote the manuscript by revising it critically for important intellectual content. PC and VL are the physicians who provided medical treatments and contributed to collected data. RB and RS gave clinical suggestions for the manuscript. PP conceived the research and gave final approval of the version to be submitted. All authors approved the final version of the manuscript and were accountable for the content of the work.

## Conflict of Interest

The authors declare that the research was conducted in the absence of any commercial or financial relationships that could be construed as a potential conflict of interest. AT declared a shared affiliation with one of the authors, RL, to the handling editor at the time of review.

## References

[B1] Aaron R. V. Fisher E. A. de la Vega R. Lumley M. A. Palermo T. M. (2019). Alexithymia in individuals with chronic pain and its relation to pain intensity, physical interference, depression, and anxiety: a systematic review and meta-analysis. *Pain* 160 994–1006. 10.1097/j.pain.0000000000001487 31009416 PMC6688175

[B2] Adams L. M. Turk D. C. (2018). Central sensitization and the biopsychosocial approach to understanding pain. *J. Appl. Biobehav. Res.* 23:e12125 10.1111/jabr.12125

[B3] Ailliet L. Rubinstein S. M. Knol D. Van Tulder M. W. de Vet H. C. (2016). Somatization is associated with worse outcome in a chiropractic patient population with neck pain and low back pain. *Man. Ther.* 21 170–176. 10.1016/j.math.2015.07.007 26254262

[B4] Apkarian A. V. Baliki M. N. Farmer M. A. (2013). Predicting transition to chronic pain. *Curr. Opin. Neurol.* 26 360–367. 10.1097/WCO.0b013e32836336ad 23823463 PMC4887742

[B5] Apkarian V. Baliki M. Geha P. (2009). Towards a theory of chronic pain. *Prog. Neurobiol.* 87 81–97. 10.1016/j.pneurobio.2008.09.018 18952143 PMC2650821

[B6] Arnold L. M. Choy E. Clauw D. J. Goldenberg D. L. Harris R. E. (2016). Fibromyalgia and chronic pain syndromes: a white paper detailing current challenges in the field. *Clin. J. Pain* 32 737–746. 10.1097/AJP.0000000000000354 27022674 PMC4974061

[B7] Asmundson G. J. G. Noel M. Petter M. Parkerson H. A. (2012). Pediatric fear-avoidance model of chronic pain: foundation, application and future directions. *Pain Res. Manag.* 17 397–405. 10.1155/2012/908061 23248813 PMC3659014

[B8] Babatunde O. O. Jordan J. L. Van der Windt D. A. Hill J. C. Foster N. E. Protheroe J. (2017). Effective treatment options for musculoskeletal pain in primary care: a systematic overview of current evidence. *PLoS One* 12:e0178621. 10.1371/journal.pone.0178621 28640822 PMC5480856

[B9] Bagby R. M. Parker J. D. Taylor G. J. (1994). The twenty-item Toronto Alexithymia Scale—I. Item selection and cross-validation of the factor structure. *J. Psychosom. Res.* 38 23–32. 10.1016/0022-3999(94)90005-1 8126686

[B10] Bagby R. M. Parker J. D. A. Taylor G. J. (2020). Twenty-five years with the 20-item Toronto Alexithymia Scale. *J. Psychosom. Res.* 131:109940. 10.1016/j.jpsychores.2020.109940 32007790

[B11] Bagby R. M. Taylor G. J. Parker J. D. A. Dickens S. E. (2006). The development of the Toronto Structured Interview for Alexithymia: item selection, factor structure, reliability and concurrent validity. *Psychother. Psychosom.* 75 25–39. 10.1159/000089224 16361872

[B12] Bailey P. E. Henry J. D. (2007). Alexithymia, somatization, and negative affect in a community sample. *Psychiatry Res.* 150 13–20. 10.1016/j.psychres.2006.05.024 17258817

[B13] Bean D. J. Johnson M. Kydd R. (2014). Relationships between psychological factors, pain, and disability in complex regional pain syndrome and low back pain. *Clin. J. Pain* 30 647–653. 10.1097/AJP.0000000000000007 24135903

[B14] Birket-Smith M. (2001). Somatization and chronic pain. *Acta Anaesthesiol. Scand.* 45 1114–1120. 10.1034/j.1399-6576.2001.450911.x 11683662

[B15] Bjelland I. Dahl A. A. Haug T. T. Neckelmann D. (2002). The validity of hospital anxiety and depression scale, An updated literature review. *J Psychosom Res.* 52 69–77. 10.1016/S0022-3999(01)00296-3 11832252

[B16] Bourke J. H. Langford R. M. White P. D. (2015). The common link between functional somatic syndromes may be central sensitisation. *J. Psychosom. Res.* 78 228–236. 10.1016/j.jpsychores.2015.01.003 25598410

[B17] Breivik H. Collett B. Ventafridda V. Cohen R. Gallacher D. (2006). Survey of chronic pain in Europe: prevalence, impact on daily life, and treatment. *Eur. J. Pain* 10 287–333. 10.1016/j.ejpain.2005.06.009 16095934

[B18] Breivik H. Eisenberg E. O’Brien T. (2013). The individual and societal burden of chronic pain in Europe: the case for strategic prioritisation and action to improve knowledge and availability of appropriate care. *BMC Public Health* 24:1229. 10.1186/1471-2458-13-1229 24365383 PMC3878786

[B19] Bressi C. Taylor G. J. Parker J. D. A. Bressi S. Brambilla V. Aguglia E. (1996). Cross-validation of the factor structure of the 20-item Toronto Alexithymia Scale: an Italian multicenter study. *J. Psychosom. Res.* 41 551–559. 10.1016/S0022-3999(96)00228-0 9032718

[B20] Bucci W. (1997). Symptoms and symbols: a multiple code theory of somatization. *Psychoanal. Inquiry* 17 151–172. 10.1080/07351699709534117

[B21] Burton A. K. Tillotson K. M. Main C. Hollis S. (1995). Psychosocial predictors of outcome in acute and sub chronic low back trouble. *Spine* 20 722–728. 10.1097/00007632-199503150-00014 7604349

[B22] Cameron K. Ogrodniczuk J. Hadjipavlou G. (2014). Changes in alexithymia following psychological intervention: a review. *Harv. Rev. Psychiatry* 22 162–178. 10.1097/HRP.0000000000000036 24736520

[B23] Castelli L. Tesio V. Colonna F. Molinaro S. Leombruni P. Bruzzone M. (2012). Alexithymia and psychological distress in fibromyalgia: prevalence and relation with quality of life. *Clin. Exp. Rheumatol.* 30(6 Suppl. 74), 70–77. 23110722

[B24] Clark J. R. Nijs J. Yeowell G. Goodwin P. (2017). What are the predictors of altered central pain modulation in chronic musculoskeletal pain populations? A Systematic Review. *Pain Phys.* 20 487–500. 28934779

[B25] Cleeland C. S. Ryan K. M. (1994). Pain assessment: global use of the brief pain inventory. *Ann. Acad. Med. Singap.* 23, 129–138. 8080219

[B26] Cohen J. (1988). *Statistical Power Analysis for the Behavioral Sciences*, 2nd Edn Abingdon: Routledge.

[B27] De Gucht V. Fischler B. Heiser W. (2004). Personality and affect as determinants of medically unexplained symptoms in primary care: a follow-up study. *J. Psychosom. Res.* 56 279–285. 10.1016/S0022-3999(03)00127-2 15046963

[B28] De Gucht V. Heiser W. (2003). Alexithymia and somatisation: quantitative review of the literature. *J. Psychosom. Res.* 54 425–434. 10.1016/s0022-3999(02)00467-1 12726898

[B29] Deyo R. A. Mirza S. K. Turner J. A. Martin B. I. (2009). Overtreating chronic back pain: time to back off? *J Am Board Fam Med.* 22 62–68. 10.3122/jabfm.2009.01.080102 19124635 PMC2729142

[B30] Di Tella M. Castelli L. (2016). Alexithymia in chronic pain disorders. *Curr. Rheumatol. Rep.* 18:41. 10.1007/s11926-016-0592-x 27215759

[B31] Di Tella M. Ghiggia A. Tesio V. Romeo A. Colonna F. (2017). Pain experience in Fibromyalgia Syndrome: the role of alexithymia and psychological distress. *J. Affect. Disord.* 208 87–93. 10.1016/j.jad.2016.08.080 27750065

[B32] Dionne C. Koepsell T. D. Von Korff M. Deyo R. A. Barlow W. E. Checkoway H. (1995). Formal education and backrelated disability. In search of an explanation. *Spine* 20 2721–2730. 10.1097/00007632-199512150-00014 8747251

[B33] Dionne C. E. Koepsell T. D. Von Korff M. Deyo R. A. Barlow W. E. Checkoway H. (1997). Predicting long-term functional limitations among back pain patients in primary care settings. *J. Clin. Epidemiol.* 50 31–43. 10.1016/s0895-4356(96)00313-7 9048688

[B34] Eliasen M. Kreiner S. Ebstrup J. F. Poulsen C. H. Lau C. J. Skovbjerg S. (2016). Somatic symptoms: prevalence, co-occurrence and associations with self-perceived health and limitations due to physical health–a Danish population-based study. *PLoS One* 11:e150664. 10.1371/journal.pone.0150664 26930630 PMC4773248

[B35] Fayaz A. Croft P. Langford R. Donaldson J. Jones G. (2016). Prevalence of chronic pain in the UK: a systematic review and meta-analysis of population studies. *BMJ Open.* 6:e010364. 10.1136/bmjopen-2015-010364 27324708 PMC4932255

[B36] Fishbain D. A. Lewis J. E. Gao J. Cole B. Steele Rosomoff R. (2009). Is chronic pain associated with somatization/hypochondriasis? An evidence-based structured review. *Pain Pract.* 9 449–467. 10.1111/j.1533-2500.2009.00309.x 19735366

[B37] Flor H. Turk D. (2011). *The Psychology Of Pain Chronic Pain: An Integrated Biobehavioral Approach.* Seattle: IASP Press, 69–80.

[B38] Friedrichsdorf S. J. Giordano J. Desai Dakoji K. Warmuth A. Daughtry C. Schulz C. A. (2016). Chronic pain in children and adolescents: diagnosis and treatment of primary pain disorders in head, abdomen, muscles and joints. *Children* 3:42. 10.3390/children3040042 27973405 PMC5184817

[B39] Gagliese L. Katz J. (2012). Medically unexplained pain is not caused by psychopathology. *Pain Res. Manag.* 5 251–257. 10.1155/2000/701397

[B40] Gandek B. Ware J. E. Aaronson N. K. Apolone G. Bjorner J. B. Brazier J. E. (1998). Cross-validation of item selection and scoring for the SF-12 Health Survey in nine countries: results from the IQOLA Project. *J. Clin. Epidemiol.* 51 1171–1178. 10.1016/s0895-4356(98)00109-7 9817135

[B41] Gatchel R. J. Peng Y. B. Peters M. L. Fuchs P. N. Turk D. C. (2007). The biopsychosocial approach to chronic pain: scientific advances and future directions. *Psychol. Bull.* 133 581–624. 10.1037/0033-2909.133.4.581 17592957

[B42] General Assembly of the World Medical Association (2014). World Medical Association Declaration of Helsinki: ethical principles for medical research involving human subjects. *J. Am. Coll. Dent.* 81:14. 25951678

[B43] Genizi J. Srugo I. Kerem N. C. (2013). The cross- ethnic variations in the prevalence of headache and other somatic complaints among adolescents in Northern Israel. *J. Head. Pain* 14:21. 10.1186/1129-2377-14-21 23566020 PMC3620373

[B44] Hayes A. F. (2013). *Introduction to Mediation, Moderation, And Conditional Process Analysis: A Regression-Based Approach.* New York, NY: Guilford Press.

[B45] Honkalampi K. Lehto S. M. Koivumaa-Honkanen H. Hintikka J. Niskanen L. Valkonen-Korhonen M. (2011). Alexithymia and tissue inflammation. *Psychother. Psychosom.* 80 359–364. 10.1159/000327583 21829048

[B46] Horta-Baas G. Peláez-Ballestas I. Queipo G. Montero Hernández U. Romero-Figueroa M. (2020). Alexithymia is associated with mood disorders, impairment in quality of life and disability in women with fibromyalgia. *Clin. Exp. Rheumatol.* 123 17–24. 31603073

[B47] Institute of Medicine (2011). *Relieving Pain in America: A Blueprint For Transforming Prevention, Care, Education, and Research.* Washington, DC: National Academies Press. 22553896

[B48] International Association for the Study of Pain (1994). *Classification of Chronic Pain: Descriptions of Chronic Pain Syndromes and Definitions of Pain Terms.* IASP Press.

[B49] International Association for the Study of Pain (2012). *Unrelieved Pain Is A Major Global Healthcare Problem.* Washington, DC: International Association for the Study of Pain.

[B50] Keefe F. Rumble M. Scipio D. Giordano L. Perri M. (2004). Psychological aspects of persistent pain: current state of the science. *J. Pain* 5 195–211. 10.1016/j.jpain.2004.02.576 15162342

[B51] Kroenke K. Spitzer R. L. Williams J. B. (2002). The PHQ-15: validity of a new measure for evaluating the severity of somatic symptoms. *Psychosom. Med.* 64 258–266. 10.1097/00006842-200203000-00008 11914441

[B52] Larice S. Ghiggia A. Di Tella M. Romeo A. Gasparetto E. Fusaro E. (2020). Pain appraisal and quality of life in 108 outpatients with rheumatoid arthritis. *Scand. J. Psychol.* 61 271–280. 10.1111/sjop.12592 31674683

[B53] Lipowski Z. J. (1988). Somatization: the concept and its clinical application. *Am. J. Psychiatry* 145 1358–1368. 10.1176/ajp.145.11.1358 3056044

[B54] Luminet O. Bagby R. M. Taylor G. J. (2018). *Alexithymia: Advances in Research, Theory, and Clinical Practice.* Cambridge: Cambridge University Press.

[B55] Lumley M. A. Neely L. C. Burger A. J. (2007). The assessment of alexithymia in medical settings: implications for understanding and treating health problems. *J. Person Ass.* 89 230–246. 10.1080/00223890701629698 18001224 PMC2931418

[B56] Lumley M. A. Stettner L. Wehmer F. (1996). How are alexithymia and physical illness linked? A review and critique of pathways. *J. Psychosom. Res.* 41 505–518. 10.1016/S0022-3999(96)00222-X 9032714

[B57] Martínez M. P. Sánchez A. I. Miró E. Lami M. J. Prados G. Morales A. (2015). Relationships between physical symptoms, emotional distress, and pain appraisal in fibromyalgia: the moderator effect of alexithymia. *J. Psychol.* 149 115–140. 10.1080/00223980.2013.844673 25511201

[B58] Mattila A. K. Kronholm E. Jula A. Salminen J. K. (2008). Alexithymia and somatization in general population. *Psychosom. Med.* 70 716–722. 10.1097/PSY.0b013e31816ffc39 18596251

[B59] McAndrew L. M. Crede M. Maestro K. Slotkin S. Kimber J. Phillips L. A. (2019). Using the common-sense model to understand health outcomes for medically unexplained symptoms: a meta-analysis. *Health Psychol. Rev.* 13 427–446. 10.1080/17437199.2018.1521730 30196755

[B60] McBeth J. Macfarlane G. J. Benjamin S. Silman A. J. (2001). Features of somatization predict the onset of chronic widespread pain: Results of a large population-based study. *Arth. Rheumat.* 44 940–946. 11315933 10.1002/1529-0131(200104)44:4<940::AID-ANR151>3.0.CO;2-S

[B61] Merskey H. Bogduk N. (1994). *Classification of Chronic Pain: Descriptions Of Chronic Pain Syndromes And Definitions Of Pain Terms*, 2nd Edn Seattle: IASP Press.

[B62] Morales-Espinoza E. M. Kostov B. Salami D. C. Perez Z. H. Rosalen A. P. Molina J. O. (2016). Complexity, comorbidity, and health care costs associated with chronic widespread pain in primary care. *Pain* 157 818–826. 10.1097/j.pain.0000000000000440 26645546

[B63] Naylor B. Boag S. Gustin S. M. (2017). New evidence for a pain personality? A critical review of the last 120 years of pain and personality. *Scand. J. Pain* 17 58–67. 10.1016/j.sjpain.2017.07.011 28850375

[B64] Nijs J. Van H. B. Oostendorp R. A. (2010). Recognition of central sensitization in patients with musculoskeletal pain: application of pain neurophysiology in manual therapy practice. *Man. Ther.* 15 135–141. 10.1016/j.math.2009.12.001 20036180

[B65] Norton S. Cosco T. Doyle F. Done J. Sacker A. (2013). The Hospital Anxiety and Depression Scale: a meta confirmatory factor analysis. *J. Psychosom. Res.* 74 74–81. 10.1016/j.jpsychores.2012.10.010 23272992

[B66] Oberleitner L. M. Lumley M. A. Grekin E. R. Smith K. Loree A. M. Carty J. N. (2019). Problematic prescription opioid use in a chronic pain treatment facility: the role of emotional processes. *Subst. Misuse* 54 495–505. 10.1080/10826084.2018.1521426 30380985

[B67] Ogrodniczuk J. S. Piper W. E. Joyce A. S. (2011). Effect of alexithymia on the process and outcome of psychotherapy: a programmatic review. *Psychiatry Res.* 190 43–48. 10.1016/j.psychres.2010.04.026 20471096

[B68] Pincus T. Burton A. K. Vogel S. Field A. P. (2002). A systematic review of psychological factors as predictors of chronicity/disability in prospective cohorts of low back pain. *Spine* 27 109–120. 10.1097/00007632-200203010-00017 11880847

[B69] Porcelli P. Guidi J. Sirri L. Grandi S. Grassi L. Ottolini F. (2013). Alexithymia in the medically ill. *Analysis of* 1,190 patients in gastroenterology, cardiology, oncology and dermatology. *Gen. Hosp. Psychiatry* 35 521–527. 10.1016/j.genhosppsych.2013.04.005 23664571

[B70] Porcelli P. Taylor J. (2018). “Alexithymia and Physical Illness: A Psychosomatic Approach,” in *Alexithymia: Advances in Research, Theory, and Clinical Practice*, eds Luminet O. Bagby R. M. Taylor G. J. (Cambridge: Cambridge University Press), 10.1017/9781108241595.011

[B71] Porcelli P. Tulipani C. Di Micco C. Spedicato M. R. Maiello E. (2011). Temporal stability of alexithymia in cancer patients following a psychological intervention. *J. Clin. Psychol.* 67 1177–1187. 10.1002/jclp.20839 22052601

[B72] Preacher K. J. Hayes A. F. (2008). Asymptotic and resampling strategies for assessing and comparing indirect effects in multiple mediator models. *Behav. Res. Methods* 40 879–891. 10.3758/BRM.40.3.879 18697684

[B73] Ratnamohan L. Kozlowska K. (2017). When things get complicated: at-risk attachment in children and adolescents with chronic pain. *Clin. Child Psychol. Psychiatry.* 22 588–602. 10.1177/1359104517692850 28994326

[B74] Reid K. J. Harker J. Bala M. M. Truyers C. Kellen E. Bekkering G. E. (2011). Epidemiology of chronic non-cancer pain in Europe: narrative review of prevalence, pain treatments and pain impact. *Curr. Med. Res. Opin.* 27 449–462. 10.1185/03007995.2010.545813 21194394

[B75] Rousseau S. Grietens H. Vanderfaeillie J. Hoppenbrouwers K. Wiersema J. R. Baetens I. (2014). The association between parenting behavior and somatization in adolescents explained by physiological responses in adolescents. *Int. J. Psychophysiol.* 93 261–266. 10.1016/j.ijpsycho.2014.05.008 24862009

[B76] Saariaho A. S. Saariaho T. H. Mattila A. K. Joukamaa M. I. Karukivi M. (2016). The role of alexithymia: an 8-year follow-up study of chronic pain patients. *Compr. Psychiatry* 69 145–154. 10.1016/j.comppsych.2016.05.015 27423355

[B77] Saariaho A. S. Saariaho T. H. Mattila A. K. Karukivi M. R. Joukamaa M. I. (2013). Alexithymia and depression in a chronic pain patient sample. *Gen. Hosp. Psychiatry* 35 239–245. 10.1016/j.genhosppsych.2012.11.011 23333032

[B78] Saariaho A. S. Saariaho T. H. Mattila A. K. Ohtonen P. Joukamaa M. Karukivi M. (2017). Alexithymia and depression in the recovery of chronic pain patients: a follow-up study. *Nord. J. Psychiatry* 71 1–8. 10.1080/08039488.2016.1275782 28413937

[B79] Sancassiani F. Machado S. Ruggiero V. Cacace E. Carmassi C. Gesi C. (2017). The management of fibromyalgia from a psychosomatic perspective: an overview. *Int. Rev. Psychiatry* 29 473–488. 10.1080/09540261.2017.1320982 28681628

[B80] Societal Impact of Pain (2017). Impact of Pain on Society Costs the EU up to 441 Billion Euros Annually.

[B81] Sirri L. Fava G. A. Sonino N. (2013). The unifying concept of illness behavior. *Psychother Psychosom.* 82 74–81. 10.1159/000343508 23295460

[B82] Stone A. L. Wilson A. C. (2016). Transmission of risk from parents with chronic pain to offspring. *Pain* 157 2628–2639.2639. 10.1097/j.pain.0000000000000637 27380502 PMC5592972

[B83] Taylor A. B. MacKinnon D. P. Tein J.-Y. (2008). Tests of the three-path mediated effect. *Org. Res. Methods.* 11 241–269. 10.1177/1094428107300344

[B84] Taylor G. J. Bagby R. M. Parker J. D. A. (1997). *Disorders of Affect Regulation, Alexithymia in Medical and Psychiatric Illness.* Cambridge: Cambridge University Press.

[B85] Tesio V. Di Tella M. Ghiggia A. Romeo A. Colonna F. Fusaro E. (2018). Alexithymia and depression affect quality of life in patients with chronic pain: a study on 205 patients with Fibromyalgia. *Front. Psychol.* 9:442. 10.3389/fpsyg.2018.00442 29670558 PMC5893813

[B86] Tulipani C. Morelli F. Spedicato M. R. Maiello E. Todarello O. Porcelli P. (2010). Alexithymia and cancer pain: the effect of psychological intervention. *Psychother. Psychosom.* 79 156–163. 10.1159/000286960 20185972

[B87] Tuzer V. Bulut S. D. Bastug B. Kayalar G. Göka E. Bes̨tepe E. (2011). Causal attributions and alexithymia in female patients with fibromyalgia or chronic low back pain. *Nord. J. Psychiatry* 65 138–144. 10.3109/08039488.2010.522596 20874000

[B88] Vlayen J. W. Linton S. J. (2012). Fear-avoidance model of chronic musculoskeletal pain: 12 years on. *Pain* 153 1144–1147. 10.1016/j.pain.2011.12.009 22321917

[B89] Vowles K. E. McEntee M. L. Julnes P. S. Frohe T. Ney J. P. van der Goes D. N. (2015). Rates of opioid misuse, abuse, and addiction in chronic pain: a systematic review and data synthesis. *Pain* 156 569–576. 10.1097/01.j.pain.0000460357.01998.f14 25785523

[B90] Waller E. Sheidt C. E. (2006). Somatoform disorders as disorders of affect regulation: a development perspective. *Int. Rev. Psychiatry.* 18 13–24. 10.1080/09540260500466774 16451876

[B91] Wiffen P. J. Xia J. (2020). Systematic review of topical diclofenac for the treatment of acute and chronic musculoskeletal pain. *Curr. Med. Res. Opin* 3 1–14. 10.1080/03007995.2020.1716703 31944135

[B92] Zigmond A. S. Snaith R. P. (1983). The hospital anxiety and depression scale. *Acta Psychiatr. Scand.* 67. 6 361–370. 10.1111/j.1600-0447.1983.tb09716.x1983.tb09716.x 6880820

